# Efgartigimod in the treatment of Guillain-Barré syndrome: case report

**DOI:** 10.3389/fimmu.2025.1586663

**Published:** 2025-09-04

**Authors:** Min Deng, Zhaohong Kong, Yan Wang, Xufeng Wang, Tao Li

**Affiliations:** ^1^ Renmin Hospital of Wuhan University, Wuhan, China; ^2^ Department of Gastrointestinal Surgery, Hubei Provincial Hospital of Traditional Chinese Medicine, Wuhan, Hubei, China

**Keywords:** Guillain-Barré syndrome, efgartigimod, Miller-Fisher syndrome, acute motor and sensory axonal neuropathy, acute inflammatory demyelinating polyneuropathy

## Abstract

Guillain–Barré syndrome (GBS) is a rare neurological disorder characterized by muscle weakness and paralysis. Although the exact etiology remains unclear, the current standard treatments include intravenous immunoglobulin (IVIG) and plasma exchange (PLEX) therapy. While the majority of GBS patients respond well to immunotherapy, some severe cases can be fatal. Efgartigimod, an Fc receptor antagonist, has been utilized in the treatment of various autoimmune diseases. However, its clinical efficacy in acute GBS has been rarely documented. In this study, we administered intravenous efgartigimod to four patients with different subtypes of acute GBS, two of whom received efgartigimod monotherapy without concomitant glucocorticoids, IVIG, or PLEX. The treatment outcomes were favorable, suggesting that intravenous efgartigimod may represent a promising therapeutic option for acute GBS. Further research is warranted to validate these preliminary findings.

## 1 Introduction

Guillain-Barré syndrome (GBS) is an acute autoimmune polyradiculoneuropathy characterized by rapidly progressive, ascending flaccid paralysis. Worldwide, approximately 100,000 people develop this disease each year. While paralysis is a prominent feature, the syndrome encompasses a spectrum of neurological deficits, including sensory disturbances, cranial nerve involvement, and dysautonomia. The exact cause of GBS is unknown. However, about two-thirds of patients have symptoms of infection in the 6 weeks before illness onset (1). Approximately 20–30% of patients with GBS may have severe GBS with respiratory failure. Globally, the mortality rate of GBS patients is approximately 7.5% (1, 2). There is no significant difference in the incidence rate of 1–2 people per 100,000 per year between genders or countries (3).

Intravenous immunoglobulin (IVIG) and plasma exchange (PLEX) therapy are considered to be the best treatments. Efgartigimod (efgartigimod alfa-fcab, Vyvgart™) is an Fc receptor antagonist used to treat autoimmune diseases including myasthenia gravis. Subcutaneous efgartigimod has been FDA- approved in the USA for treatment of Chronic Inflammatory Demyelinating Polyradiculoneuropathy (CIDP), and that two more Fc receptor (FcRn) antagonists, rozanolixizumab and nipocalimab are also approved for generalized AchR and MuSK antibody positive myasthenia gravis (4). There are several ongoing clinical studies of efgartigimod for the treatment of other autoimmune diseases, including bullous pemphigoid, chronic inflammatory demyelinating polyradiculoneuropathy, immune thrombocytopenia, and autoimmune myositis. However, the clinical efficacy of efgartigimod in acute GBS has been documented in only a limited number of cases (5–7). In this study, we administered efgartigimod to four patients with different subtypes of acute GBS. Among them, two patients received efgartigimod as monotherapy, one was treated with a combination of IVIG and efgartigimod, and the remaining patient received glucocorticoids alongside efgartigimod. Notably, all four patients achieved favorable clinical outcomes, highlighting the potential therapeutic benefits of efgartigimod in acute GBS management.

## 2 Methods

### 2.1 Research design and data collection

Data from the current cases were systematically collected and analyzed to comprehensively evaluate the clinical characteristics, disease progression, and treatment responses associated with this condition. To identify factors influencing clinical outcomes, detailed analyses were performed on demographic information, prodromal symptoms, clinical manifestations, disease severity at peak and last follow-up, diagnostic findings, treatment strategies, and clinical outcomes.

Disease severity was assessed at peak disability and during each follow-up visit using standardized scales, including the Medical Research Council Scale (MRC) for muscle strength, the Guillain-Barré Syndrome Disability Scale (GBS-DS) for functional disability, and the Inflammatory Neuropathy Cause and Treatment Disability Scale (INCAT) for limb functionality. These scales provided a quantitative and qualitative framework to monitor disease progression and therapeutic efficacy.

In addition, electromyography and nerve conduction studies were performed before and after drug treatment to objectively evaluate changes in nerve conduction velocity, amplitude, and latency. These electrophysiological assessments served as critical biomarkers to complement clinical evaluations and further validate the effectiveness of the treatment interventions. Furthermore, longitudinal follow-up data were collected to assess long-term outcomes, including residual disability, quality of life, and potential complications.

## 3 Case presentations

### 3.1 Case 1

A 51-year-old female patient began to develop fever and cough in January 2024. One week later, she developed bilateral eyelid ptosis, accompanied by dyspnea and limb weakness. She was admitted to a community hospital and received antiviral treatment, but her symptoms did not resolve, so she attended our hospital and was admitted. She had a history of hypertension. She reported no history of diabetes, infectious diseases (such as hepatitis B and tuberculosis), surgical trauma, or poisoning, and no family history. Neurological examination revealed bilateral ptosis and ophthalmoplegia with the absence of light reflex. She could not show her teeth or puff her cheeks and she had loss of nasolabial folds and loss of forehead wrinkles, suggesting facial diplegia. Muscle strength in all four limbs was graded as Medical Research Council grade 4. The tendon reflexes in both the upper and lower extremities were diminished.

Nerve conduction studies demonstrated reduced motor nerve conduction velocity (MNCV) in the right median nerve. Compound motor action potential (CMAP) amplitudes were at the lower normal limits bilaterally in the median and ulnar nerves. Reduced sensory nerve action potential (SNAP) amplitudes with decreased sensory nerve conduction velocity (SNCV) were observed in bilateral median and ulnar nerves. Reduced CMAP amplitudes were noted in bilateral facial nerves. The blink reflex study revealed complete absence of R1, R2, and R2’ waveforms on the left side with sparse R1 and R2 waveforms on the right. Needle EMG detected minimal abnormal spontaneous activity in the left first dorsal interosseous muscle.

Cerebrospinal fluid (CSF) analysis showed normal intracranial pressure (130 mmH_2_O), normal leukocytes (3×10^6^), normal protein (0.28 g/L), and normal glucose (3.8 mmol/L). The blood gas analysis results were as follows: pH of 7.32, oxygen partial pressure of 90 mmHg, carbon dioxide partial pressure of 55 mmHg, bicarbonate level of 26.0 mmol/L, and standard bicarbonate level of 28.0 mmol/L. Liver function, blood lipids, blood cell count, kidney function, procalcitonin, respiratory etiology indicators, electrolytes, blood glucose, myocardial infarction indicators, and coagulation function were normal. Chest X-ray, electrocardiogram, and cardiac color ultrasound were normal.

Peripheral neuropathy-associated antibodies in CSF and serum (GM1, GM2, GM3, GM4, GD1a, GD1b, GD2, GD3, GT1a, GT1b, GQ1b, sulfatide, NF155, NF186, CNTN1, CNTN2, CASPR1, and MAG) were assessed using immunofluorescence staining. No antibodies were detected in the CSF. GM2 IgM, GM1 IgM, and sulfatide IgM were positive in the serum. The patient was diagnosed with Miller–Fisher syndrome and Guillain–Barré overlap syndrome (MFS GBS overlap syndrome).

The patient received IVIG (0.4 g/kg/day for 5 days), ventilator-assisted respiratory support, and efgartigimod (10 mg/kg via weekly intravenous infusion for 3 weeks). She was discharged after dyspnea and peripheral facial paralysis improved.

**Figure 1:**
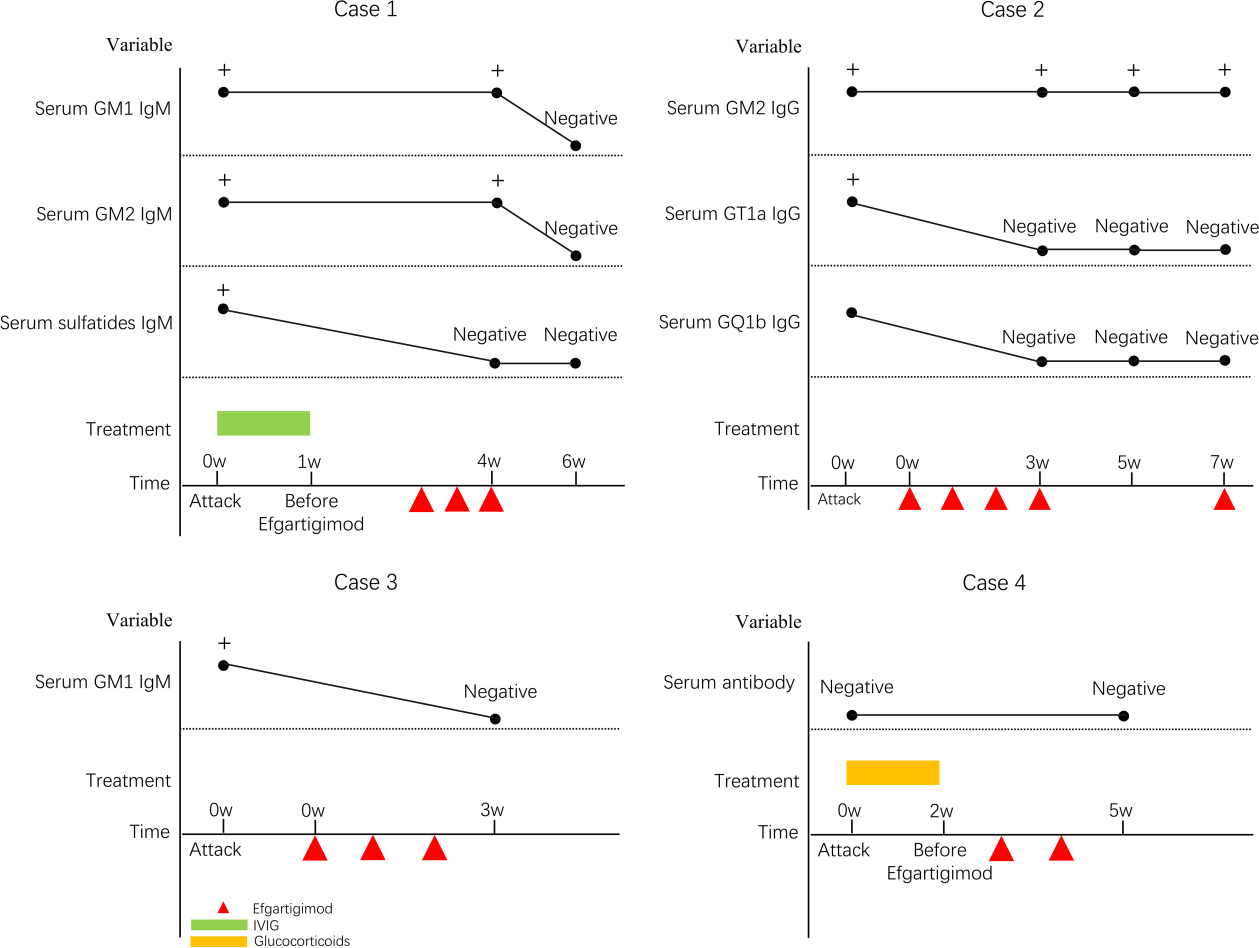
Changes in serum antibodies.

**Figure 2:**
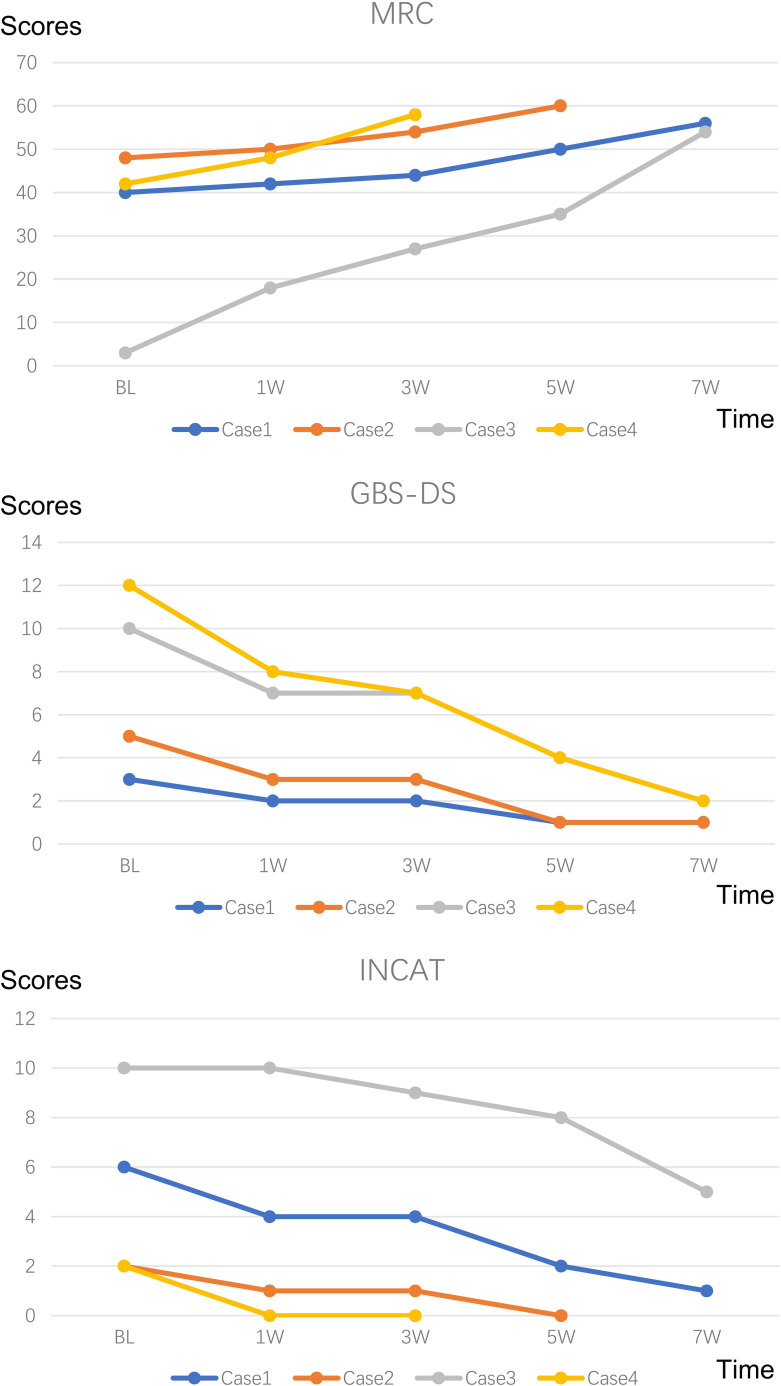
Changes in MRC, GBS-DS and INCAT score. MRC Sum Score: Quantifies muscle strength by summing scores from six bilateral limb muscles (three upper/three lower limbs), ranging from 60 (normal) to 0 (quadriplegia); GBS-DS: Functional disability scale graded 0-6 (0=healthy; 6=death); INCAT: Assesses limb functionality through validated mobility and arm function subscales.

Her extraocular movements and bilateral eyelid elevation had recovered, and she had no symptoms of unsteady gait at her follow-up examination 2 months after treatment. No adverse drug reactions occurred. The clinical manifestations, diagnosis, treatments, and follow-up of all four patients are detailed in **Tables 1**–**3** and **Figures 1**, **2**.

**Table 1:** Clinical manifestations and diagnosis of cases.

Details	Case1	Case2	Case3	Case4
Age	51y	42y	30y	36y
Gender	Woman	Woman	Man	Man
Symptom duration before diagnosis	3d	10d	2d	13d
Symptom	fever, cough, bilateral eyelid ptosis, bilateral eyeball inactivity, dyspnea and limbs weakness	fever and cough, bilateral eyeball inactivity, blurred vision and unsteady walking	weakness in his limbs, dyspnea	limbs weakness and dysphagia
Neurological signs	peripheral facial paralysis, the limbs strength was level 4	eyeballs fixed, gag reflex weakened, finger-nose test positive	limbs strength is grade 0, the tendon reflex and gag reflex disappear	peripheral facial paralysis, the limbs strength was level 4, the tendon reflexes were weak
CSF	leukocytes (3×10^6^), protein (0.28g/l)	leukocytes (2×10^6^), protein (0.3g/l)	leukocytes (2×10^6^), protein (0.32g/l)	leukocytes (4×10^6^), protein (1.46g/l)
Serum antibodies	GM1 IgM (+) GM2 IgM (+) sulfatides IgM (+)	GM2 IgG (+) GT1a IgG (+) GQ1b IgG (+)	GM1 IgM (+)	Negative
Types of GBS	MFS GBS overlap syndrome	MFS	AMSAN	AIDP
Doses of Efgartigimod	3	5	3	2
IVIG	Yes	–	–	–
Glucocorticoids	–	–	–	Yes
Ventilator	Yes	–	Yes	–
Initial MRC	40	48	3	42
Initial GBS-DS	3	2	5	2
Initial INCAT	6	2	10	2

MFS, Miller–Fisher Syndrome.

GBS, Guillain–Barré Syndrome.

AMSAN, Acute Motor and Sensory Axonal Neuropathy.

AIDP, Acute Inflammatory Demyelinating Polyneuropathy.

IVIG, Intravenous Immunoglobulin.

MRC, Medical Research Council.

GBS-DS, GBS Disability Score.

INCAT, Inflammatory Neuropathy Cause and Treatment.

**Table 2:** Changes of motor nerve conduction before and after treatment.

Site	Case 1						Case 2						Case 3						Case 4		
	1^st^ Study (January 24, 2024)			2^nd^ Study (February 5, 2024)			1^st^ Study (February 2, 2024)			2^nd^ Study (February 28, 2024)			1^st^ Study (March 11, 2024)			2^nd^ Study (April 1, 2024)			1^st^ Study (March 7, 2024)		
	Latency, ms	Amplitude, mV	Velocity, m/s	Latency, ms	Amplitude, mV	Velocity, m/s	Latency, ms	Amplitude, mV	Velocity, m/s	Latency, ms	Amplitude, mV	Velocity, m/s	Latency, ms	Amplitude, mV	Velocity, m/s	Latency, ms	Amplitude, mV	Velocity, m/s	Latency, ms	Amplitude, mV	Velocity, m/s
Left median (Abductor pollicis brevis)																					
Wrist	3.30	7.3		3.11	7.4		3.5	10.1		3.50	10.4		3.00	**4.4↓**		2.88	6.1		**5.42↑**	7.1	
Elbow	7.64	7.1	51.8	7.13	7.3	54.7	7.83	10.7	52	7.75	10.1	57.6	6.63	**3.7↓**	60.6	6.75	5.8	64.6	10.1	7.1	**48.1↓**
Right median (Abductor pollicis brevis)																					
Wrist	3.24	7.4		3.41	8.7		3.17	8.5		2.88	8.1		2.95	**2.4↓**		3.00	4.3		**8.83↑**	**4.3↓**	
Elbow	7.94	7.7	**48.9↓**	7.28	8.4	62	7.42	7	51.8	7.17	8.0	57.1	7.47	**2.3↓**	53.1	7.42	3.8	50.9	13.8	**4.5↓**	**48.3↓**
Left ulnar (Abductor digiti minimi)																					
Wrist	1.98	7.3		2.08	7.3		2.38	10.0		2.13	9.8		2.38	**3.5↓**		2.13	7.1		**5.05↑**	**5.9↓**	
Below elbow	7.04	7.0	57.8	7.29	6.2	54.6	8.86	7.1	**41.3↓**	7.45	8.5	51.3	7.96	**4.3↓**	58.5	7.88	6.3	51.1	10.9	**5.5↓**	59.1
Right ulnar (Abductor digiti minimi)																					
Wrist	1.87	6.9		2.29	7.4		2.17	10		2.17	9.9		unobtained	unobtained		unobtained	unobtained		2.92	8.1	
Below elbow	6.92	6.2	67.6	6.92	8.1	62.1	7.5	7.0	52.5	7.5	8.4	52.5	unobtained	unobtained	unobtained	unobtained	unobtained	unobtained	8.43	7.6	64.9
Left peroneal (Extensor digitorum brevis)																					
Ankle	2.96	4.6		3.04	4.4		3.88	5.5		3.71	4.8		3.5	**1.19↓**		**5.13↑**	**0.14↓**		**5.47↑**	3.5	
Below Fibular	10.00	4.8	42.6	9.25	4.3	49.9	10.2	4.7	47.5	9.38	4.5	52.0	9.54	**1.24↓**	44.7	10.9	**0.21↓**	46.8	12.5	3.0	46.9
Right peroneal (Extensor digitorum brevis)																					
Ankle	3.71	5.2		2.63	7.3		3.96	4.0		3.65	3.6		unobtained	unobtained		**5.37↑**	3.0		**6.13↑**	2.4	
Below Fibular	9.92	5.2	47.5	8.71	6.8	51	10.2	3.9	50.5	9.67	4.2	49.8	unobtained	unobtained	unobtained	unobtained	unobtained	unobtained	13.8	2.4	41.7
Left tibial (Abductor hallucis brevis)																					
Ankle	3.25	14.8	unobtained	3.05	17.6	unobtained	3.79	9.3	unobtained	4.08	11.1	unobtained	3.85	**1.67↓**	unobtained	4.46	**0.33↓**	unobtained	4.51	**2.5↓**	unobtained
Right tibial (Abductor hallucis brevis)																					
Ankle	3.33	18.7	unobtained	2.73	17.1	unobtained	3.58	8.1	unobtained	4.67	11.1	unobtained	3.79	7.6	unobtained	4.00	9.7	unobtained	4.29	**2.8↓**	unobtained
Left oculi (Orbicularis oculi)																					
Mastoid	2.57	**0.89↓**	unobtained	2.78	**1.63↓**	unobtained	3.21	2.3	unobtained	2.78	3.1	unobtained	unobtained	unobtained	unobtained	unobtained	unobtained	unobtained	unobtained	unobtained	unobtained
Right oculi (Orbicularis oculi)																					
Mastoid	3.34	**0.56↓**	unobtained	2.43	**1.51↓**	unobtained	3.21	2.7	unobtained	3.15	3.2	unobtained	unobtained	unobtained	unobtained	unobtained	unobtained	unobtained	unobtained	unobtained	unobtained

The bold values indicate statistical significance. The symbols "↓" and "↑" denote a decrease and increase, respectively.

**Table 3:** Changes of sensory nerve conduction before and after treatment.

Site	Case 1						Case 2						Case 3						Case 4		
	1^st^ Study (January 24, 2024)			2^nd^ Study (February 5, 2024)			1^st^ Study (February 2, 2024)			2^nd^ Study (February 28, 2024)			1^st^ Study (March 11, 2024)			2^nd^ Study (April 1, 2024)			1^st^ Study (March 7, 2024)		
	Latency, ms	Amplitude, μV	Velocity, m/s	Latency, ms	Amplitude, μV	Velocity, m/s	Latency, ms	Amplitude, μV	Velocity, m/s	Latency, ms	Amplitude, μV	Velocity, m/s	Latency, ms	Amplitude, μV	Velocity, m/s	Latency, ms	Amplitude, μV	Velocity, m/s	Latency, ms	Amplitude, μV	Velocity, m/s
Left median anti-sensory (3rd digit)																					
Wrist	2.15	**16.2↓**	58.1	2.19	**19.3↓**	61.6	2.95	**6.7↓**	**42.4↓**	2.38	23.0	52.5	1.92	28.9	75.5	2.11	48.5	61.6	unobtained	unobtained	unobtained
Right median anti-sensory (3rd digit)																					
Wrist	2.15	**12.7↓**	65.1	2.29	**16.2↓**	59	3.09	**7.0↓**	**42.1↓**	2.03	22.7	59.1	2.18	32.9	64.2	2.04	28.6	68.6	unobtained	unobtained	unobtained
Left radial anti-sensory (base 1st digit)																					
Wrist	unobtained	unobtained	unobtained	unobtained	unobtained	unobtained	1.44	**16.6↓**	66.0	1.54	32.9	64.9	1.71	**10.0↓**	40.9	2.29	**3.6↓**	48.0	1.52	**12.8↓**	62.5
Right radial anti-sensory (base 1st digit)																					
Wrist	1.55	29.6	71	1.57	33.9	66.9	1.37	**14.7↓**	69.3	1.3	41.8	73.1	1.73	**5.8↓**	46.2	2.35	**3.2↓**	46.8	1.67	**14.8↓**	68.9
Left ulnar anti-sensory (5th digit)																					
Wrist	1.69	**11.7↓**	62.1	1.76	**20.6↓**	54	2.48	**5.4↓**	**44.4↓**	1.64	**16.0↓**	61.0	1.81	**14.1↓**	55.2	1.70	21.9	64.7	unobtained	unobtained	unobtained
Right ulnar anti-sensory (5th digit)																					
Wrist	1.9	**15.2↓**	57.9	1.86	**15.1↓**	51.1	1.94	**2.5↓**	51.5	1.58	21.3	60.1	unobtained	unobtained	unobtained	unobtained	unobtained	unobtained	2.19	**3.3↓**	50.2
Left superficial peroneal anti-sensory (Lat Mall)																					
Leg	1.9	32.8	57.9	1.77	28.1	50.8	2.02	**7.7↓**	44.6	1.96	**8.5↓**	40.8	unobtained	unobtained	unobtained	unobtained	unobtained	unobtained	1.82	**6.3↓**	49.5
Left superficial peroneal anti-sensory (Lat Mall)																					
Leg	1.78	24.6	56.2	1.44	34.3	62.5	1.75	**7.3↓**	51.4	1.68	**8.8↓**	53.6	unobtained	unobtained	unobtained	unobtained	unobtained	unobtained	2.33	**4.7↓**	47.2
Left sural anti-sensory (Lat Mall)																					
Calf	1.77	14.4	59.3	1.51	15.5	59.6	1.74	19.8	57.5	1.37	17.4	58.4	2.02	6.8	47.0	unobtained	unobtained	unobtained	1.84	13.1	51.6
Right sural anti-sensory (Lat Mall)																					
Calf	1.54	16	58.4	1.63	15.1	55.2	1.96	14.9	43.4	1.75	20.8	51.4	1.69	16.6	44.4	2.23	13.8	49.3	2.23	15.0	51.6
Left median F-wave	27.5	39.5		27.6	17.4		26.9	262		25.1	310		25.7	6.6		24.2	337		**34.8↑**	196	
Right median F-wave	27.8	15.9		27.8	267		26.6	211		25.3	199		25.5	13.3		24.9	227		**20.8↑**	181	
Left tibial F-wave	50.7	590		51	355		47.0	182		45.0	416		unobtained	unobtained		unobtained	unobtained		44.5	142	
Right tibial F-wave	50.7	385		48.5	274		48.6	0.231		46.2	0.617		45.4	0.146		43.5	0.468		43.4	0.108	
Left ulnar F-wave	26.7	171		26.5	143		27.0	0.336		25.3	0.424		unobtained	unobtained		26.6	0.213		**31.8↑**	0.229	
Right ulnar F-wave	26.3	185		26.3	213		27.5	0.178		25.2	0.350		unobtained	unobtained		unobtained	unobtained		25.7	0.377	

The bold values indicate statistical significance. The symbols "↓" and "↑" denote a decrease and increase, respectively.

### 3.2 Case 2

A 42-year-old female patient began to develop fever and cough in January 2024. One week later, she developed bilateral complete opthalmoplegia, blurred vision, and unsteady walking. She received antiviral treatment at a community hospital, but her symptoms did not resolve, so she attended our hospital and was admitted. She reported no history of chronic diseases (such as hypertension and diabetes), infectious diseases (such as hepatitis B and tuberculosis), surgical trauma, or poisoning, and no family history. Neurological examination showed that she could not move her eyeballs bilaterally, her gag reflex was weakened, and she exhibited instability in the finger-to-nose and finger-to-finger tests. She exhibited normal tendon reflexes in upper and lower extremities.

Nerve conduction studies demonstrated decreased MNCV in the left ulnar nerve, reduced SNAP amplitudes bilaterally in median, ulnar, radial, superficial peroneal and tibial nerves, and decreased SNCV bilaterally in median and tibial nerves with left ulnar involvement; in the blink reflex study, prolonged latencies and low amplitudes were observed for bilateral R1/R2 waveforms. On needle EMG, minimal abnormal spontaneous activity was detected in the left first dorsal interosseous. CSF analysis showed normal intracranial pressure (155 mmH_2_O), normal leukocytes (2×10^6^), normal protein (0.3 g/L), and normal glucose (3.2 mmol/L). Liver function tests showed elevated alanine aminotransferase (143.00 U/L) and aspartate aminotransferase (120.00 U/L). Lipid tests showed elevated cholesterol (5.65 mmol/L), triacylglycerol (3.14 mmol/L), and low-density lipoprotein cholesterol (3.61 mmol/L). Blood cell count, kidney function, procalcitonin, respiratory etiology indicators, blood gas analysis results, electrolytes, blood glucose, myocardial infarction indicators, and coagulation function were normal. Head and spinal cord magnetic resonance imaging (MRI) scans, chest computed tomography (CT) scan, electrocardiogram, and cardiac color ultrasound were normal.

Peripheral neuropathy-associated antibodies in CSF and serum were assessed using immunofluorescence staining. No antibodies were detected in the CSF. GM2 IgG, GT1a IgG, and GQ1b IgG were positive in the serum. The patient was diagnosed with Miller–Fisher syndrome (MFS).

She received five doses of Efgartigimod (10 mg/kg intravenous infusion), administered weekly for the first four doses, followed by a fifth dose one month later. She was discharged after her eyeball movements returned to normal.

She showed no gait instability at the 7-week follow-up, but serum GM2 IgG antibodies remained positive. No adverse drug reactions occurred. The clinical manifestations, diagnosis, treatments, and follow-up of all four patients are detailed in **Tables 1**–**3** and **Figures 1**, **2**.

### 3.3 Case 3

A 30-year-old male patient attended our hospital and was admitted in February 2024 due to sudden slurred speech and difficulty breathing. A CT scan of his head showed brainstem bleeding, and he received symptomatic treatment, including treatment to control his blood pressure. He developed sudden weakness in his limbs 25 days after hospitalization. He reported no history of chronic diseases (such as hypertension and diabetes), infectious diseases (such as hepatitis B and tuberculosis), surgical trauma, or poisoning, and no family history. Neurological examination showed grade 0/5 strength in both proximal and distal muscles of the upper and lower limbs and absent tendon reflexes.

Nerve conduction studies revealed: Unelicitable CMAP in the right ulnar nerve; Reduced CMAP amplitudes bilaterally in median and ulnar nerves, with left tibial and peroneal nerve involvement; Unelicitable SNAP in superficial peroneal nerves; Reduced SNAP amplitudes bilaterally in radial nerves, and in left ulnar/sural nerves; Absent F-waves bilaterally in ulnar nerves and left tibial nerve. On needle EMG, significant abnormal spontaneous activities were observed in: bilateral first dorsal interosseous, extensor digitorum communis, tibialis anterior, gastrocnemius (medial heads), quadriceps (vastus lateralis), and left biceps brachii.

CSF analysis showed normal intracranial pressure (130 mmH_2_O), normal leukocytes (2×10^6^), normal protein (0.32 g/L), and normal glucose (3 mmol/L). Liver function, blood lipids, blood cell count, kidney function, procalcitonin, electrolytes, and blood glucose were normal. A head CT scan showed bleeding in the pons. Chest CT scan, electrocardiogram, and cardiac color ultrasound were normal.

Peripheral neuropathy-associated antibodies in CSF and serum were assessed using immunofluorescence staining. No antibodies were detected in the CSF. GM1 IgM was positive in serum. The patient was diagnosed with acute motor and sensory axonal neuropathy (AMSAN).

He received ventilator-assisted respiratory therapy, anti-infective treatment (ceftazidime), and efgartigimod (10 mg/kg via weekly intravenous infusion for 3 weeks). When muscle strength in both upper and lower limbs reached grade 3 (MRC scale), the patient was transferred to the rehabilitation department for continued therapy.

At the 6 months follow-up assessment, muscle strength had recovered to grade 5/5 in all extremities. No adverse drug reactions occurred. The clinical manifestations, diagnosis, treatments, and follow-up of all four patients are detailed in **Tables 1**–**3** and **Figures 1**, **2**.

### 3.4 Case 4

A 36-year-old male patient suddenly developed limb weakness and dysphagia without any obvious cause in February 2024. He attended our hospital 4 days later. He reported no history of hypertension, diabetes, infectious diseases, surgical trauma, or poisoning, and no family history. Neurological examination showed droopy bilateral eyelids, shallow bilateral frontal lines and nasolabial folds, weak gag and tendon reflexes, and grade 4/5 strength in both proximal and distal muscles of the upper and lower limbs.

Nerve conduction studies demonstrated: Prolonged distal motor latencies with reduced CMAP amplitudes in bilateral median nerves (right amplitude reduction) and left ulnar nerve; Reduced CMAP amplitudes in bilateral tibial nerves; Prolonged motor distal latencies in bilateral common peroneal nerves; Unelicitable SNAPs in bilateral median and left ulnar nerves; Reduced SNAP amplitudes in bilateral radial, superficial peroneal, and right ulnar nerves; Prolonged F-wave latencies in bilateral median and left ulnar nerves. In the blink reflex study, markedly prolonged latencies were observed for bilateral R1 (Reference value 10–13 ms) and R2 (Reference value 30–40 ms) waveforms.

CSF analysis showed normal intracranial pressure (140 mmH_2_O), normal leukocytes (4×10^6^), elevated protein (1.46 g/L), and normal glucose (3.5 mmol/L). These findings are consistent with albuminocytological dissociation. Liver function, blood lipids, blood cell count, kidney function, procalcitonin, electrolytes, and blood glucose were normal. Head MRI scan, chest CT scan, electrocardiogram, and cardiac color ultrasound were normal.

Peripheral neuropathy-associated antibodies in CSF and serum were assessed using immunofluorescence staining. No antibodies were detected in the CSF or serum. The patient was diagnosed with acute inflammatory demyelinating polyneuropathy (AIDP).

Due to the combined pressures of financial constraints and treatment accessibility challenges, both IVIG and PLEX were declined by the patient and their family. After being informed of the risks associated with corticosteroid therapy, they provided written consent for a course of methylprednisolone treatment (initial 500mg dose tapered by half every 3 days over 15 days). As this proved ineffective, the patient subsequently received efgartigimod (10 mg/kg via weekly intravenous infusion for 2 weeks).

The peripheral facial palsy resolved completely, accompanied by restoration of muscle strength to MRC grade 5/5 in all four extremities. No adverse drug reactions occurred. The clinical manifestations, diagnosis, treatments, and follow-up of all four patients are detailed in **Tables 1**–**3** and **Figures 1**, **2**.

## 4 Discussion

Our case series demonstrates that efgartigimod therapy is effective and safe across GBS variants, with sustained clinical improvement observed in all subtypes. GBS is the most common cause of acute flaccid paralysis worldwide.

Diagnosis of typical cases is usually straightforward. However, current diagnostic criteria have limitations that may result in missing atypical cases. Biomarkers that are used to diagnose typical cases may not apply to most GBS variants (8). One of the four cases was antibody negative, but his clinical symptoms supported a diagnosis of AIDP and he had increased protein in his CSF with a normal number of cells. In this study, normal CSF protein levels were observed in 3 out of 4 patients, which may be attributable to the early timing of lumbar puncture. All procedures were performed within the first week of admission, prior to the typical peak of albuminocytological dissociation. The higher antibody positivity rate (75%) may reflect the small cohort size (n=4) rather than true population prevalence, as limited samples can magnify statistical variability.

The detection of IgM anti-GM1 and anti-GM2 antibodies in case 1 patient with MFS-GBS overlap syndrome represents a departure from the classic serological profile of isolated MFS, which is dominated by IgG anti-GQ1b antibodies. While these IgM antibodies are typically associated with acute motor axonal neuropathy or motor-predominant GBS, their presence here may reflect an expanded autoimmune response targeting shared ganglioside epitopes within peripheral nerve complexes. Mechanistically, molecular mimicry between GM1/GM2 and GQ1b-containing glycolipid clusters could permit cross-reactive binding, while the potent complement-activating capacity of IgM may exacerbate axonal injury aligning with our electrophysiological findings of reduced CMAP amplitudes and prolonged distal latencies. Although causality cannot be definitively established, the temporal correlation of peak antibody titers with progressive limb weakness (exceeding typical MFS) supports a potential contributory role in disease pathogenesis, consistent with prior reports of anti-GM antibodies amplifying motor deficits in GBS. Further studies profiling ganglioside-specific IgM in overlap syndromes are warranted to validate their pathophysiological significance. Approximately one quarter of GBS patients require artificial ventilation (9). Many patients develop autonomic disorders and their symptoms typically peak within 4 weeks, followed by a weakening of the immune response and repair of peripheral nerves (10). The recovery period from this disease can last months or years.

Most people with GBS do well with immunotherapy, but some severe cases can be fatal. Additionally, most people are able to walk again 6 months after symptoms first appear, but some people remain disabled (1). Therefore, it is crucial that patients receive treatment as soon as possible, especially patients with rapidly progressive GBS.

Conventional treatments for acute GBS include IVIG and PLEX. At present, there is no clear understanding of how IVIG inhibits harmful inflammatory reactions, but it is generally believed to be related to the inhibition of Fc receptors. Beyond FcRn saturation mediated IgG half-life extension, IVIG’s anti-idiotypic antibodies directly neutralize pathogenic autoantibodies by sterically blocking their binding to neural targets. Subsequently, the formation of immune complexes facilitates accelerated clearance of circulating autoantibodies via FcγRIIB-dependent phagocytosis (11, 12). Concurrently, IVIG disrupts the complement cascade at critical junctures: specifically, it inhibits C3 convertase assembly to prevent amplification, while simultaneously binding C5b-7 complexes to obstruct membrane attack complex deposition on nerve membranes. Neonatal FcRn binds to IgG and protects it from lysosomal degradation. Efgartigimod is a IgG1 Fc fragment with strong affinity for FcRn (stronger than that of regular IgG), allowing it to compete with IgG for FcRn binding and reduce IgG recycling. Of the four cases, one was treated with IVIG and efgartigimod, one with glucocorticoids and efgartigimod, and two with efgartigimod only. Effective recovery of neurological function was observed in all cases. No drug-related adverse reactions occurred in any case. We regularly measured pathogenic antibodies in serum to assess the efficacy and safety of efgartigimod. After several efgartigimod injections, the pathogenic antibodies became negative in three cases, while serum GM2 IgG antibodies remained in one case.

This study, along with three previous case reports, evaluates the efficacy of efgartigimod in GBS, collectively supporting its potential as an effective and safe novel therapeutic option. The literature includes three pivotal case reports demonstrating efgartigimod’s therapeutic potential across neurological autoimmune conditions. Zhou et al. documented a 58-year-old AMAN patient with suboptimal response to IVIg/PLEX who achieved significant clinical and electrophysiological improvement after once-weekly infusions (10 mg/kg) over 4 weeks, establishing its efficacy in refractory axonal GBS (6). Zhang et al. reported two GBS cases where accelerated dosing (two 10 mg/kg doses within 5 days) enabled independent ambulation by 4 weeks, highlighting rapid functional recovery with transient adverse events (5). Watanabe et al. described an 84-year-old with anti-GQ1b syndrome overlapping myasthenia gravis, in whom efgartigimod induced ventilator weaning within 7 days and restored mobility after conventional therapies failed, proving its value in complex, treatment-resistant disorders (7). In contrast to prior single-case reports describing combination therapy (administered following failure of IVIG or PLEX), the current work presents a case series (n=4). Notably, it includes two monotherapy cases (without concurrent IVIG or PLEX), encompasses diverse GBS subtypes, demonstrates acute-phase application, and reports well-defined favorable treatment outcomes.

## 5 Conclusion

The rapid elimination of pathogenic antibodies is crucial for the effective treatment of GBS. Efgartigimod, an FcRn antagonist, accelerates antibody clearance by inhibiting the recycling of IgG into the bloodstream. Four patients treated with intravenous efgartigimod exhibited favorable clinical outcomes, suggesting its ability to rapidly reduce GBS-related antibodies and alleviate symptoms. These findings indicate that efgartigimod may be a promising therapeutic option for acute GBS. Further studies are needed to confirm its broader applicability.

## Data Availability

The original contributions presented in the study are included in the article/Supplementary Material. Further inquiries can be directed to the corresponding authors.
